# Detection of Antimicrobial Resistance Genes in the Milk Production Environment: Impact of Host DNA and Sequencing Depth

**DOI:** 10.3389/fmicb.2020.01983

**Published:** 2020-08-26

**Authors:** Selene Rubiola, Francesco Chiesa, Alessandra Dalmasso, Pierluigi Di Ciccio, Tiziana Civera

**Affiliations:** Department of Veterinary Sciences, University of Turin, Turin, Italy

**Keywords:** antimicrobial resistance genes, whole metagenome sequencing, milk, host DNA depletion, sequencing depth

## Abstract

Over the past decades, antimicrobial resistance (AMR) has been recognized as one of the most serious threats to public health. Although originally considered a problem to human health, the emerging crisis of AMR requires a “One Health” approach, considering human, animal, and environmental reservoirs. In this regard, the extensive use of antibiotics in the livestock production systems to treat mastitis and other bacterial diseases can lead to the presence of AMR genes in bacteria that contaminate or naturally occur in milk and dairy products, thereby introducing them into the food chain. The recent development of high-throughput next-generation sequencing (NGS) technologies is improving the fast characterization of microbial communities and their functional capabilities. In this context, whole metagenome sequencing (WMS), also called shotgun metagenomic sequencing, allows the generation of a vast amount of data which can be interrogated to generate the desired evidence, including the resistome. However, the amount of host DNA poses a major challenge to metagenome analysis. Given the current absence of literature concerning the application of WMS on milk to detect the presence of AMR genes, in the present study, we evaluated the effect of different sequencing depths, host DNA depletion methods and matrices to characterize the resistome of a milk production environment. WMS was conducted on three aliquots of bulk tank milk and three aliquots of the in-line milk filter collected from a single dairy farm; a fourth aliquot of milk and milk filter was bioinformatically subsampled. Two commercially available host DNA depletion methods were applied, and metagenomic DNA was sequenced to two different sequencing depth. Milk filters proved to be the most suitable matrices to evaluate the presence of AMR genes; besides, the pre-extraction host DNA depletion method was the most efficient approach to remove host reads. To our knowledge, this is the first study to evaluate the limitations posed by the host DNA in investigating the milk resistome with a WMS approach, confirming the circulation of AMR genes in the milk production environment.

## Introduction

The increasing prevalence of antimicrobial resistance (AMR) in bacteria is one of the most serious threats to public health (Schrijver et al., 2018). Latest data report that AMR is responsible for more than 30,000 deaths per year in the European Union (EU) and European Economic Area (EEA) and costs about 1.1 billion euros to the health care systems of EU/EEA countries (Boolchandani et al., 2019). Although originally considered a problem to human health, the emerging crisis of AMR requires a one health approach, considering human, animal, and environmental reservoirs (Van Puyvelde et al., 2018). In this regard, of particular concern is the high use of antibiotics in livestock production systems, as it is known that the emergence and selection of resistant bacteria can be associated to the antimicrobial administration due to ecological pressure. From a public health perspective, the possible transmission of resistance bacteria from food-producing animals to humans is considered a major risk; indeed, the increasing presence of AMR bacteria in farm animals can lead to their dissemination to humans *via* direct contact with livestock or *via* the food chain (Call et al., 2008).

The AMR spread may occur through different mechanisms; in fact, the genetic dynamics of AMR include multiple strategies, from point mutations to the exchange of entire genes. Among the mechanisms behind its occurrence, the presence of AMR genes on horizontally transmissible elements can lead to their transmission to new bacterial hosts, both pathogenic and commensal, pointing out the risk to human health (Schrijver et al., 2018). The recent advances in next-generation sequencing (NGS) technologies and bioinformatic analyses have brought continuous advantages in this area, improving fast AMR genes identification and characterization (Boolchandani et al., 2019). Culture-based resistance determination involves the isolation of target bacteria and the assessment of the growth in the presence of antimicrobials; taking into account the large amount of unculturable bacteria, this method underestimates species and AMR genes. On the other hand, culture-independent methods have many potential advantages; among them, whole metagenome sequencing (WMS), also called shotgun metagenomic sequencing, is accomplished by unrestricted sequencing of the genomes of all the microorganisms within a sample, including uncultured ones, allowing the generation of a large amount of data which can be interrogated to evaluate the presence of AMR genes (Zaheer et al., 2018).

Though overcoming the intrinsic bias of culture-dependent and amplicon-based methods, the huge potential of WMS brings some challenges (Addis et al., 2016). For the analysis of host-derived samples, in fact, the advantage of sequencing the total DNA extracted from a sample becomes also a vulnerability, as a consequence of the predominance of host DNA; saliva, blood, and milk are just few examples of the host-derived sample which usually contain more than 90% of host genome-aligned reads (Marotz et al., 2018; McHugh et al., 2020). This kind of sample requires the application of molecular and bioinformatics tools to remove the unwanted host DNA; besides, a deep sequencing depth is critical to obtain a reasonable coverage of the microbial genomes (Pereira-Marques et al., 2019). A greater sequencing depth increases the number of generated sequences but, at the same time, can quickly escalate the costs of sequencing, reducing the possibility of application on a large number of samples (Thoendel et al., 2016). Actually, few studies have investigated the impact of this limitation on the sensitivity of WMS to gain a complete picture of the microbiome of host-derived samples (Zaheer et al., 2018; Gweon et al., 2019; Pereira-Marques et al., 2019; Heravi et al., 2020). In this regard, a very recent publication dealt with this issue investigating the dairy microbiome (McHugh et al., 2020); the authors’ conclusions highlighted how, due to the high proportion of *Bos taurus* reads sequenced, the resulting low number of microbial reads did not allow a strain-level classification or an in-depth functional analysis, addressing the possible use of a microbiome enrichment kit to overcome the problem for future analyses.

There are several commercial kits available to remove host DNA, acting prior to DNA extraction (pre-extraction methods) or after DNA extraction (post-extraction methods). Among them, MolYsis Basic5 kit (Molzym, Bremen, Germany) takes advantage of differential lysis of mammals and microbial cells, lysing eukaryotic cells through a chaotropic reagent and removing the released DNA by enzymatic digestion prior to extraction of bacterial DNA. On the other hand, among post-extraction methods, the NEBNext Microbiome DNA Enrichment Kit (New England BioLabs, Ipswich, MA) selectively bounds and removes CpG-methylated host DNA, taking advantage of the low CpG methylation rates of microbial species. Both techniques have been used in different studies on human samples (Thoendel et al., 2016; Marotz et al., 2018; Heravi et al., 2020) and in few works on animal stool samples (Thomas et al., 2017); however, none of them have ever been applied on food matrices, such as milk. Very little is known about the required sequencing depth necessary to investigate complex samples. Though fully capturing all DNA sequences from the microbiome of a complex matrix is not practical, despite continuous improvements in NGS technologies, estimating the required sequencing depth needed to characterize a particular microbial population is important to achieve the goal of a given study, such as the investigation of the resistome in a food production environment (Zaheer et al., 2018).

In the actual context of growing AMR concern, tracking the emergence and prevalence of AMR is a priority. Although recent research points toward the possibility of the transmission of AMR *via* the food chain and toward the relevance of food-processing environments as reservoirs of AMR, little is known about the resistome associated with dairy cattle production (Call et al., 2008; Zaheer et al., 2018; Alexa Oniciuc et al., 2020). As previously mentioned, antibiotics are used extensively in dairy farms, leading to the introduction of AMR genes in milk and dairy products (Freitag et al., 2017; Nobrega et al., 2018). In this context, the increasing preference for raw milk, which has already proven to pose a threat for the consumer, due to the possible presence of foodborne pathogens (Claeys et al., 2013), is even more troubling. Investigation of the presence of AMR genes in the milk production environment may provide precious data to estimate the public health risk associated with antibiotic usage in dairy industry.

Given the current paucity of literature concerning the application of WMS on milk to detect the presence of AMR genes, here, we carried out a pilot study to investigate the impact of varying sequencing depths, host DNA depletion methods, and milk production environment matrices on the ability to gain valuable data about the microbiome and the resistome of a milk production environment.

## Materials and Methods

### Sample Collection

**Figure 1:**
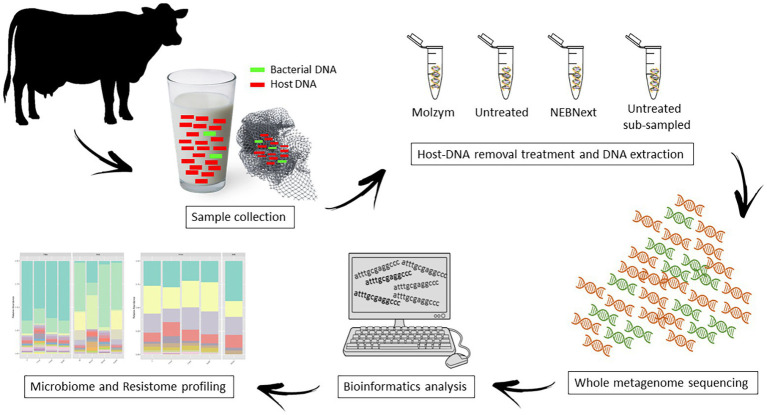
The overall workflow in this study. Bulk tank milk and in-line filter samples were divided into four aliquots each, and the total DNA was extracted using four different methods, including two microbial enrichment protocols and two different sequencing depth. Whole metagenome sequencing (WMS) was performed to investigate the microbiome and the resistome.

An aliquot of 50 ml of bulk tank milk and the disposable in-line milk filter were collected from a dairy farm located in Piedmont, North-West Italy; the selected farm had 200 lactating cows and a mean daily milk production volume of 5,000 L. Samples were collected directly from the tank in sterile conditions, inserted in sterile plastic containers, and transferred to the Laboratory of Food Inspection at the Department of Veterinary Science – University of Turin. Upon arrival, 90 ml of sterile ringer solution (Oxoid Ltd., Basingstoke, Hampshire, UK) was added to a 10 g of aseptically weighed milk filter in a sterile stomacher bag and homogenized with a Stomacher® 400 circular (Seward Ltd., Worthing, UK) at 230 rpm for 2 min. The bulk milk (sample M) and the obtained filter homogenate (sample F) were divided into three aliquots of 15 ml each (M1, M2, M3; F1, F2, F3), which were subjected to different treatment and sequencing depth; aliquots were processed as shown in Figure 1.

### Sample Treatment and DNA Extraction

Sample M1 and sample F1 were treated immediately after collection using the MolYsis Basic5 kit (Molzym, Bremen, Germany) as pre-extraction method to remove host DNA; samples were processed according to the manufacturer’s instructions. After removal of MolDNase A, DNA was extracted using the DNeasy Blood and Tissue Kit (QIAGEN, Hilden, Germany); DNA was eluted using 100 μl of 10 mM Tris-HCl buffer (pH 8.5) and frozen at −20°C before further analyses.

Samples M2, F2, M3, and F3 were extracted using the DNeasy Blood and Tissue Kit (QIAGEN, Hilden, Germany) without a pre-extraction treatment; a few modifications were applied to the manufacturer’s instructions. The overnight step was preceded by two centrifugation steps: samples were centrifuged at 100 × *g* for 10 min to pellet and discard eukaryotic cells; secondly, prokaryotic cells were pelleted from milk serum by centrifugation at 13,000 × *g* for 15 min at 4°C and pellets were resuspended in phosphate-buffered saline (Oxoid). Isolation of genomic DNA was then performed following the conventional protocol; DNA was eluted using 100 μl of 10 mM Tris-HCl buffer (pH 8.5). Samples M2 and F2 were used as untreated samples and frozen at −20°C before further analyses.

Samples M3 and F3 were treated using the NEBNext Microbiome DNA Enrichment Kit (New England BioLabs, Ipswich, MA) as post-extraction method to remove host DNA, following the manufacturer’s protocol; after the treatment, Agencourt AMPure XP beads (Beckman Coulter, Brea, CA) were used to purify the DNA following the NEBNext Microbiome DNA Enrichment Kit’s instructions. DNA was eluted using 100 μl of 10 mM Tris-HCl buffer (pH 8.5) and frozen at −20°C before further analyses.

### Whole Metagenome Sequencing

The DNA concentration of each sample was quantified using a Qubit 2.0 Fluorometer (Life Technologies, Darmstadt, Germany) with the Qubit dsDNA HS Assay Kit. DNA integrity was evaluated by agarose gel electrophoresis and purity of the DNA was determined by measuring the ratios of absorbance at 260/280 using a NanoDrop spectrophotometer (ThermoFisher Scientific) considering indicative of DNA purity a 260/280 ratio between 1.8 and 2.0. Samples meeting quality criteria were submitted for library preparation and metagenomic DNA sequencing.

DNA library preparation, WMS, and quality control steps were performed by the genomics service company Genewiz (Leipzig, Germany). NEBnext Ultra II DNA library preparation kit and four PCR cycles were applied to generate libraries (New England Biolabs, Ipswich, MA); samples were run on an Illumina NovaSeq 6000 platform to generate 2 × 150 bp paired-end (PE) reads. The required sequencing depth was 120 million PE reads for untreated samples M2 and F2 (1x), 60 million PE reads for samples M1 and F1 (0.5x), and 60 million PE reads for samples M3 and F3 (0.5x).

### Data Analysis

#### Data Pre-processing: Subsampling, Quality Filtering and Host-DNA Filtering

In order to simulate the effect of sequencing at 1/2th depth (0.5x), the two FASTQ files with the forward and reverse PE reads generated by the WMS of samples M2 and F2 were randomly subsampled bioinformatically with Seqtk tool^1^, resulting in two new samples (M4; F4) consisting of 0.5x dataset coverage.

Sequencing data quality control of the eight samples was carried out using the FastQC (version 0.11.5) with the default parameters^2^. Quality filtering of raw sequencing data was performed using Trimmomatic (version 0.33; Bolger et al., 2014) as follows: the leading and trailing low quality (below quality 3) or ambiguous base calls from sequence reads were removed; quality score filtering was performed using a sliding window at every four bases with a minimum Phred score of 15; and sequences with <50 nucleotides were discarded. Adapters were removed using a maximum of two mismatches in the initial seed and clipped if a match score of 30 was reached.

After the quality filtering step, raw reads were mapped against both a bovine reference genome (*B. taurus* ARS-UCD1.2 from NCBI genome database) and a human reference genome (*Homo sapiens* GRCh38.p13) with Bowtie2 (version 2.2; Langmead and Salzberg, 2012) using the default values, in order to identify and remove host DNA sequences and reads derived from possible human DNA contamination. The unmapped reads were then used for the downstream analysis.

#### Microbiome Analysis

The host-filtered reads were mapped against the MiniKraken2 v1 reference database built from the RefSeq bacterial and archeal libraries, using the ultrafast metagenomic classification package Kraken 2 (Wood et al., 2019) with a filtering threshold of 0.05 to enhance accuracy of taxonomic assignments; reports of the taxonomic classification of reads were generated using Kraken’s report function. Abundance estimates were generated with the package Bracken (Lu et al., 2017), which performs a Bayesian re-estimation of abundance after classification with Kraken 2.

#### Resistome Analysis

The host-filtered reads were provided as input to BWA MEM alignment (Li, 2013) using default parameters including a mismatch penalty value of 4 to the MEGARes AMR genes database (Lakin et al., 2017). Reads were assigned to AMR genes using a 75% gene coverage threshold (Zaheer et al., 2018). The BAM files generated were processed using ResistomeAnalyzer (Lakin et al., 2017). Counts of short reads aligned to the AMR genes were recorded and used for downstream analyses. Read counts originating from alignments to housekeeping genes associated with AMR present in the MEGARes database requiring single nucleotide polymorphism (SNP) confirmation were filtered out from the AMR report before further analyses.

#### Microbiome and Resistome Comparison

For microbiome and resistome comparison among sample datasets generated with different sequencing depths and treatments, sets of unique taxa for each samples were determined for each taxonomic levels (phylum, class, family, order, genus, and species) and for each AMR classification level (class, mechanism, group, and gene) for non-rarefied data and plotted as Venn diagrams. Samples without identified AMR genes were removed from further resistome analyses.

Taxonomic profiles were visualized based on the hierarchical clustering of the Bray-Curtis distances in the application Calypso (Zakrzewski et al., 2017). Rarefaction analysis was performed to compare resistome richness and coverage at different taxon levels using the RarefactionAnalyzer tool from the AMRPlusPlus pipeline (Lakin et al., 2017) with 5% subsampling increments of the reads data and 10 iterations at each level. The rarefaction curves were plotted as numbers of unique mechanisms, classes, gene groups, and genes.

## Results

### Whole Metagenome Sequencing

After the quality control process, including the evaluation of the DNA concentration, integrity, and purity, samples M1 and M3, corresponding to milk aliquots treated with the MolYsis Basic5 kit (Molzym, Bremen, Germany) and the NEBNext Microbiome DNA Enrichment Kit (New England BioLabs, Ipswich, MA), revealed a DNA concentration lower than 2 ng/μl. Nevertheless, all eight sample were submitted for library preparation and metagenomic DNA sequencing.

Processing of Illumina NovaSeq 6000 metagenomic sequencing data at different sequencing depth produced a total of 452 million reads, over 67 gb across all eight samples. For samples M1 and F1, 51 million reads were produced, while 113 and 124 million reads were produced for samples M2 and F2, respectively, and 55 and 58 million reads were produced for samples M3 and F3, respectively; finally, 56 and 62 million reads were bioinformatically subsampled for samples M4 and F4, respectively (Table 1). PCR duplication rates ranged between 8.5 and 10.6%. The mean read quality score for samples ranged from 34 to 36.

**Table 1:** Metagenomic sequencing data before and after trimming, quality filtering, and host DNA removal.

Sample ID	Sequencing coverage	Treatment	Raw reads	After quality trimming/filtering (%)	Host DNA (%)	Final reads
M1	60 M PE	MolYsis	51,025,201	68.2	82.5	6,104,646
M2	120 M PE	Untreated	112,962,895	97.9	99.4	645,372
M3	60 M PE	NEBNext	54,526,194	97	99.3	368,920
M4	60 M PE	Untreated	56,481,447	97.9	99.4	322,543
F1	60 M PE	MolYsis	51,856,657	96.6	24.7	37,759,063
F2	120 M PE	Untreated	124,198,751	98.1	91.5	10,396,311
F3	60 M PE	NEBNext	58,018,631	97.9	87.3	7,264,000
F4	60 M PE	Untreated	62,099,375	98.1	91.5	5,194,879

The table shows the number of original (raw) reads, the percentage of reads remaining after trimming and filtering, the percentage of host DNA, and the final number of reads used for the downstream analysis per sample.

### Data Analysis

#### Data Pre-processing

The reads generated survived quality filtering and trimming in different percentage depending on the type of sample and treatment. In particular, while for samples F1, F2, F3, F4, M2, M3, and M4, only 2–6% were discarded, for sample M1, corresponding to the aliquot of milk pre-treated with the MolYsis Basic5 kit (Molzym, Bremen, Germany), over 32% of reads were discarded (Table 1).

Of the total quality-filtered reads, the percentage of reads associated with the bovine genome ranged from 24.7 to 99.4% among the eight samples (Table 1). In particular, the host DNA content was >99% for samples M2, M3, and M4, while 82.5% of reads were mapped against the host DNA for sample M1, corresponding to the milk aliquot treated with the MolYsis Basic5 kit (Molzym, Bremen, Germany). As shown in Table 1, filter homogenate aliquots had a lower percentage of reads associated with the bovine genome; in particular, the host DNA content was around 90% for samples F2, F3, and F4, while 24.7% of reads were mapped against the host DNA for sample F1, corresponding to the filter aliquot treated with the MolYsis Basic5 kit (Molzym, Bremen, Germany). Human DNA sequences represented a negligible proportion in all the samples.

#### Microbiome Comparison

**Figure 2:**
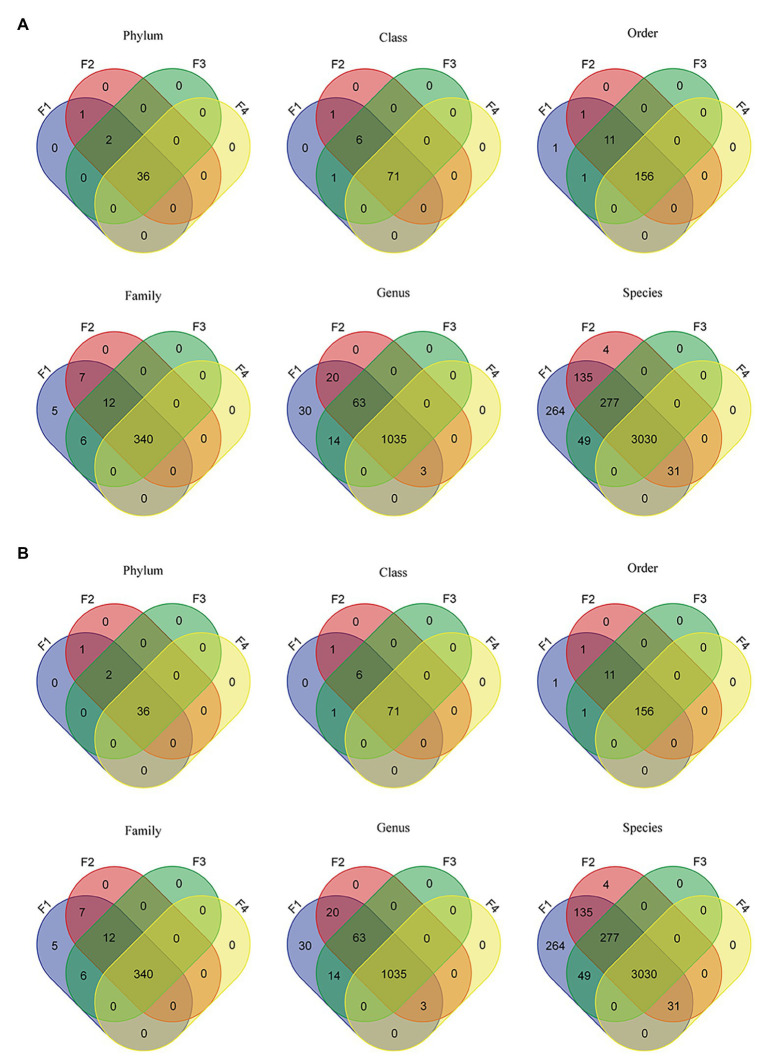
Venn diagrams representing the intersection of various microbiome taxonomic levels between filter aliquots datasets **(A)** and milk aliquots **(B)**.

Of a total 571 million reads across all datasets, 23.5 million reads (4.1%) were identified at the bacterial and archeal phyla level. As shown by Venn diagrams (Figure 2A), for filter aliquots, F1 and F2 datasets reads were assigned to the same 39 bacterial and archaeal phyla; whereas 38 and 36 phyla were identified in sample F3 and F4, respectively. For milk aliquots (Figure 2B), M1 datasets reads were assigned to 35 bacterial and archeal phyla, all included in the 39 phyla identified in filter aliquots, whereas 18, 17, and 15 of the 35 phyla were identified in samples M2, M3, and M4, respectively. Similarly, 71 classes, 156 orders, 340 families, 1,035 genera, and 3,030 species were shared between all filter aliquots, while 22 classes, 58 orders, 101 families, 134 genera, and 90 species were shared in all milk aliquots.

For filter aliquots, at all taxonomic levels, more taxa were discovered in F1 and F2 aliquots, corresponding to the filter aliquot treated with the MolYsis kit and sequenced to a depth of 60 M PE reads and to the untreated filter aliquot sequenced to a depth of 120 M PE reads; at lower taxonomic levels, including genus and species, more taxa were discovered in F2 aliquot. For milk aliquots, at all taxonomic levels, more taxa were discovered in M1 aliquot, corresponding to the milk aliquot treated with the MolYsis kit and sequenced a depth of 60 M PE reads. Considering the sequencing depth, for both milk and filter aliquots, at each taxonomic levels, more taxa were detected in the untreated aliquots sequenced to a depth of 120 M PE reads than in the untreated aliquots sequenced to a depth of 60 M PE reads (Figure 2).

**Figure 3:**
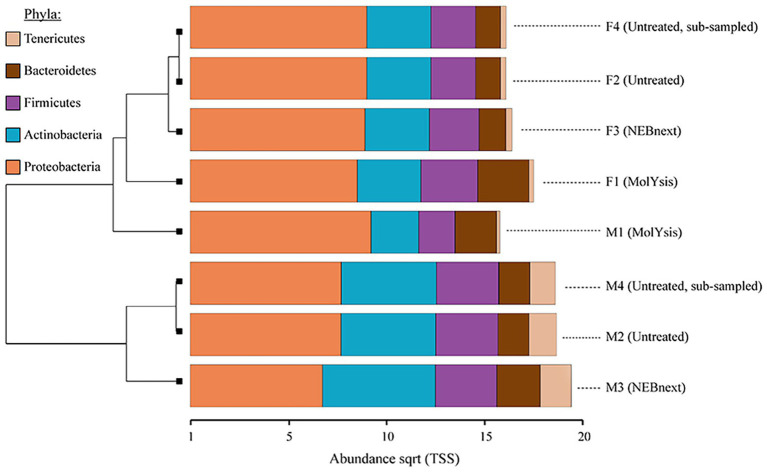
Clustered bar chart based on hierarchical clustering of the Bray-Curtis distances of milk and filter aliquots visualized in Calypso after total sum scaling (TSS) normalization. The most common bacterial phyla obtained from different sample type, sequencing depth, and microbiome DNA enrichment methods are shown. The horizontal axis is the square root abundances of the identified taxa.

The relative abundance of reads assigned to the major bacterial phyla Proteobacteria, Actinobacteria, Firmicutes, and Bacteroidetes remained similar in filter and milk aliquots; on the other hand, the actual proportion was distinctly different. In particular, focusing on the two matrices processed, more reads were assigned to bacterial phyla in filter aliquots than in milk aliquots; besides, focusing on the different treatment, among both filter and milk aliquots, more reads were assigned to bacterial phyla in aliquots treated with MolYsis Kit (F1 and M1, respectively) despite a reduced sequencing depth compared to untreated aliquots. Aliquots treated with NEBNext had the most similar bacterial pattern to the untreated aliquots for both milk and filter; this was also supported by hierarchical clustering, which clustered the taxonomic profiles of these two methods together, while untreated aliquots and MolYsis formed a separate cluster (Figure 3).

**Figure 4:**
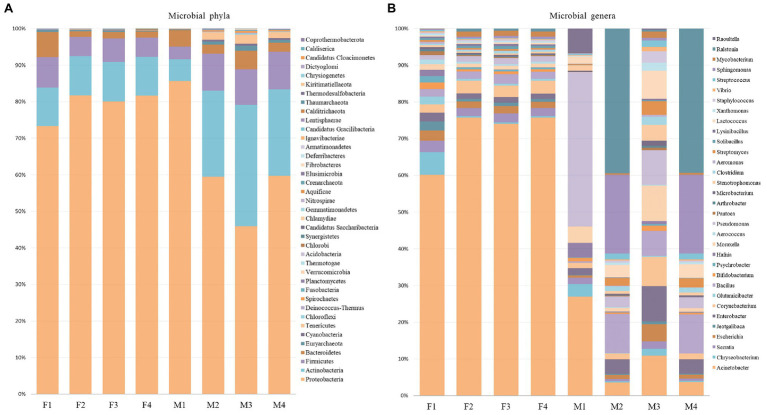
Relative proportion of microbial phyla and relative abundance of most abundant genera in filter **(A)** and milk **(B)** aliquots.

Even though the phylum-level distribution did not indicate major shifts in the bacterial community, in-depth analysis at genera-level suggested differences of the microbial profile between filter and milk aliquots, and, among them, between different treatments. As shown in Figure 4, *Acinetobacter* was the most abundant genus in filter aliquots (ranging from 51 to 64% in the different aliquots), while *Pseudomonas* in treated aliquots and *Ralstonia* in untreated aliquots were the most abundant genera in milk samples (approximately 37 and 20%, respectively). Other abundant genera in both filter and milk aliquots were *Bacillus*, *Serratia*, *Moraxella*, *Corynebacterium*, *Enterobacter*, and *Lactococcus*, with an overall prevalence of Gram-negative bacteria. Among filter aliquots, untreated aliquots with different sequencing depth and aliquot treated with NebNext kit had a similar distribution of the most abundant genera, with *Acinetobacter* (62–64%), *Corynebacterium* (3%), *Bacillus* (2%), *Serratia* (2%), *Escherichia* (2%), *Pseudomonas* (1–2%), and *Enterobacter* (1–2%) figuring among the most abundant genera with similar percentage. Few differences were detected in aliquots treated with MolYsis kit, with a lower percentage of *Acinetobacter* (51%) and a higher percentage of *Chryseobacterium* (5%), while other genera percentage remained similar. Among milk aliquots, untreated aliquots with different sequencing depth had a similar distribution of the most abundant genera, with *Ralstonia* (19–29%), *Actinoalloteichus* (16%), *Sphingomonas* (10%), *Sphingobium* (6%), *Bacillus* (5%), *Enterobacter* (2%), *Acinetobacter* (2%), and *Lactococcus* (2%) figuring among the most abundant genera. Aliquots treated with enrichment kits revealed a different distribution of the microbial population, with a higher percentage of *Pseudomonas*, *Acinetobacter*, *Raoultella*, and *Moraxella* in M1 aliquot (aliquot treated with MolYsis kit) and a higher percentage of *Cutibacterium*, *Acinetobacter*, *Moraxella*, and *Pseudomonas* in M3 aliquot (aliquot treated with NebNext kit), while lower genera percentage remained similar (Figure 4).

#### Resistome Comparison

Of the host-filtered reads, approximately 130,000 reads (0,02% of total reads) were found to be associated with the MEGARes AMR genes database across F1, F2, F3, F4, and M1 datasets; instead, no reads pass the gene fraction threshold to be considered AMR gene-associated hits in M2, M3, and M4 milk aliquots.

**Figure 5:**
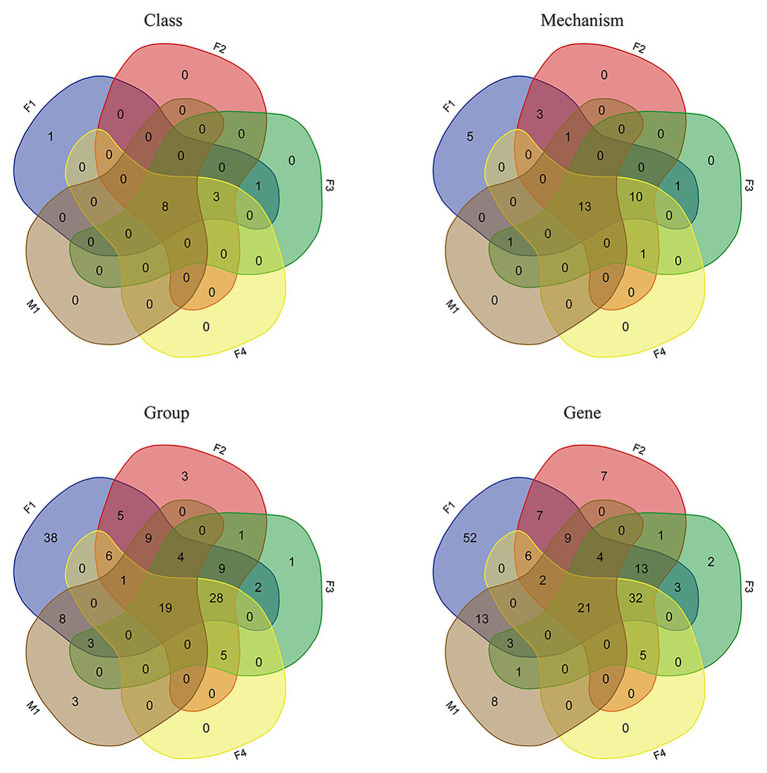
Venn diagrams representing the intersection of various classification levels of the resistome between datasets obtained in all filter aliquots and M1 milk aliquot.

As shown by Venn diagrams (Figure 5), eight AMR classes were identified in all filter aliquots and in M1 milk aliquot; these included β-lactams; tetracyclines; sulfonamides; fosfomycin; aminoglycosides; multi-drug resistance; rifampin; and macrolide, lincosamide, and streptogramin (MLS). Besides, five more AMR classes were identified in filter aliquots, including cationic antimicrobial peptides, trimethoprim, phenicol, bacitracin, and glycopeptides.

At the lower AMR annotation levels, more AMR determinants were detected in filter aliquots. Indeed, 23 AMR mechanisms, 47 AMR groups, and 53 AMR genes were shared between all filter aliquots but were absent in the milk aliquot; in F1 aliquot, corresponding to the aliquot of filter treated with MolYsis kit, 11 additional AMR mechanisms, 85 more AMR groups, and 113 more AMR genes were identified. Considering the sequencing depth, while the same number of AMR classes were detected in F2 and F4 aliquots, corresponding to the untreated aliquots sequenced to a depth of 120 M PE reads and 60 M PE reads, respectively, for lower classification levels, more mechanisms and genes were detected in F2 aliquot (Figure 5).

**Figure 6:**
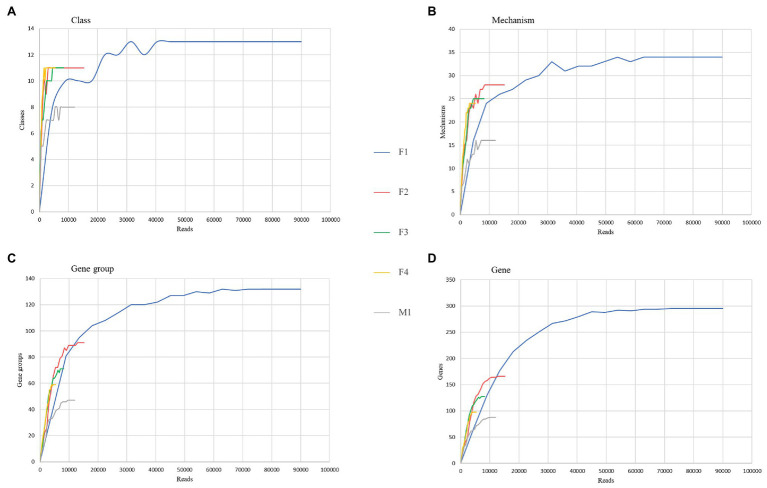
Comparison of resistome richness and coverage at the AMR class **(A)**, mechanism **(B)**, group of genes **(C)**, and gene **(D)** levels using rarefaction curves.

Rarefaction curves were generated to assess the saturation of samples for various AMR categories. As shown in Figure 6, all aliquots reached the asymptote or started to plateau up to the mechanism level; at the gene group and individual gene levels, only F1, F2, and M1 rarefaction curves reached their asymptotes, suggesting that saturation in sequencing was achieved.

Similar to microbiome results, while the relative abundance of reads assigned to the major AMR determinants remained similar in filter aliquots, the actual proportion was distinctly different. Among both filter and milk aliquots, more reads were assigned to AMR determinants in aliquots treated with MolYsis kit (F1 and M1 aliquots); in particular, among milk aliquots, no reads pass the gene fraction threshold to be considered AMR gene-associated hits in M2, M3, and M4 aliquots. Focusing on the two matrices processed, more reads were assigned to AMR determinants and more classes, mechanisms, and genes were identified in filter aliquots than in milk aliquots.

**Figure 7:**
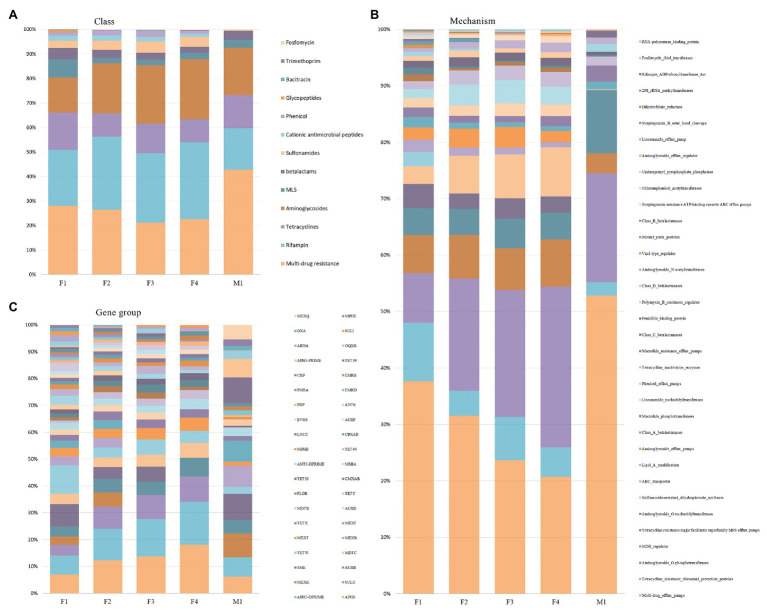
Relative abundance of AMR classes **(A)**, mechanisms **(B)**, and gene groups **(C)** across filter aliquots and M1 milk aliquot.

As shown in Figure 7A, the relative proportion of sequences associated with different AMR classes remained similar in filter aliquots; multi-drug resistance (MDR; 21–28%), rifampin (23–30%), aminoglycosides (14–24%), tetracyclines (9–15%), sulfonamides (3–5%), MLS (3–7%), and β-lactams (3–5%) were the most prevalent AMR classes. The multidrug efflux pump mechanism was most abundant (21–38%) followed by aminoglycoside O-phosphotransferase (10–28%), tetracycline resistance ribosomal protection protein (5–8%), MDR regulator (7–8%), and sulfonamide-resistant dihydropteroate synthases (3–9%). APH(6) and APH(3’) were the most prevalent amynoglicoside resistance genes identified, followed by tetracycline resistance genes belonging to TetW group, multi-drug efflux pump genes belonging to SME group, and Sulfonamide-resistant genes belonging to SULII group (Figure 7B). Few differences in the relative abundance of reads associated with different AMR determinants were relieved in M1 milk aliquot; multi-drug resistance (43%) was the most abundant AMR class, followed by aminoglycosides (19%), rifampin (17%), tetracyclines (14%), β-lactams (4%), and MLS (3%). As for filter aliquots, multidrug efflux pump mechanism was the most abundant mechanism (53%) followed by aminoglycoside O-phosphotransferase (18%) and tetracycline resistance major facilitator superfamily (MFS) efflux pumps (10%). Tetracycline resistance genes belonging to Tet39 group and multi-drug efflux pump genes belonging to SME group and MexK, MexB, and MexF groups were the most prevalent resistance genes identified (Figure 7C).

## Discussion

As a result of the rapid evolution of NGS technologies, a wide range of approaches is now available for the characterization of microbial communities in food matrices; however, the whole metagenomic DNA needs to be extracted and sequenced to gain useful information on the AMR genes content (Addis et al., 2016). Previous NGS studies have investigated the milk microbiota by 16S metabarcoding and functional metagenomics (Oikonomou et al., 2012; Addis et al., 2016; Alexa Oniciuc et al., 2020), while literature references on the application of shotgun metagenomics sequencing are limited to the evaluation of the microbiota in human milk (Ward et al., 2013; Jiménez et al., 2015). Thus, here, we compared the effectiveness of two different matrices, bulk milk and milk filter, two commercially available bacterial enrichment kits, and two different sequencing depths to investigate the microbiome and the resistome of a milk production environment with a WMS approach.

The low relative abundance of bacterial DNA presents a major challenge when using WMS to investigate the microbiome and the resistome in host-derived samples (Thoendel et al., 2016). This concept can also be applied to food matrices of animal origin, such us milk and dairy products, often resulting in more than 90% of host genome-aligned reads (Marotz et al., 2018). In our study, the milk sample and the in-line milk filter revealed a different proportion of host DNA, with a higher percentage of reads associated to the bovine genome in milk aliquots than in filter aliquots, regardless of sequencing depth and enrichment treatment. As shown in previous studies, high amounts of host DNA reduce the sensitivity of WMS on taxonomic and AMR gene content profiling, in particular for low abundant species of bacteria and AMR determinants (Thoendel et al., 2016; Pereira-Marques et al., 2019). This is consistent with the results of our study, in which more taxa and more AMR determinants were detected in filter than in milk aliquots at each classification level; in particular, only one milk aliquot revealed the presence of AMR genes, highlighting the significant effect of a massive amount of host DNA on the resistome profiling.

Considering the sequencing depth, the deep sequencing of 120 M PE reads revealed more microbial taxa than the 0.5x sequencing, in both milk and filter untreated aliquots; similar to microbiome results, less AMR determinants were detected in the sub-sampled filter aliquot, except for the highest AMR annotation level. The significant effect of sequencing depth on the characterization of the microbiome and the resistome has been demonstrated in previous studies applied on different matrices, such as environmental samples and stool samples (Zaheer et al., 2018; Gweon et al., 2019). In our study, the results obtained in milk and milk filter are consistent with previous works, though referring to food and food associated matrices. The AMR gene content and low abundant species of bacteria appeared to require a high sequencing depth to be fully characterized in untreated samples. However, the lowest sequencing depth applied appeared to be as performing as the highest sequencing depth on aliquots treated with microbial DNA enrichment methods.

In this context, we compared the effectiveness of two commercially available kits in removing the host DNA. Whether testing milk or milk filters, both techniques reduced the percentage of bovine genome associated reads; however, the MolYsis kit was much more effective than the NEBNext kit, with a reduction in percentage of host associated reads from 99.4 to 82.5% and from 91.5 to 24.7% in milk and filter aliquots, respectively. As expected, the reduction of host DNA was associated with a higher detection of microbial taxa and AMR determinants; in particular, among milk aliquots, AMR genes were detected only in the aliquot treated with MolYsis kit. Aliquots treated with NEBNext kit revealed a minor reduction of the host associated reads, with few differences in terms of AMR determinants and microbial taxa identified compared to untreated aliquots. The promising results of MolYsis kit on milk and milk filters are consistent with some limited studies regarding its application on clinical human samples, from blood to saliva, periodontal samples, and synovial and cerebrospinal fluids samples (Gebert et al., 2008; Horz et al., 2008, 2010; McCann and Jordan, 2014; Hasan et al., 2016; Thoendel et al., 2016; Ivy et al., 2018). On the other hand, the limited effectiveness of NEBNext kit has been described in previous studies on clinical human samples, probably due to the requirement of high molecular weight intact DNA input (Thoendel et al., 2016; Heravi et al., 2020). In our study, the initial centrifugation steps applied to milk aliquots and the filter homogenization might have caused shearing of high molecular weight DNA, limiting the efficacy of NEBNext kit (Heravi et al., 2020).

As any steps of sample processing and DNA isolation, enrichment methods can introduce some biases: Indeed, bacteria with weak or absent cell walls could be lysed and removed by these protocols, with the subsequent generation of an extracted DNA which does not accurately reflect the genomic content of the microbial community from which it was derived. Though microbial DNA enrichment kits have been applied on different clinical samples, the effect of this approach on mixed microbial communities in terms of the loss of microbial DNA has been scarcely investigated (Heravi et al., 2020); besides, there is a lack of literature on the application of these methods on food matrices. In our study, at higher classification levels, bacterial community composition of filter and milk aliquots was similar between untreated and enriched DNA aliquots (Figure 3). Based on the WMS analysis, Proteobacteria, Actinobacteria, Firmicutes, and Bacteroidetes phyla accounted for most of the taxon assigned OTUs identified in all the aliquots, while multi-drug resistance, rifampin, aminoglycosides, and tetracyclines were the most abundant AMR classes. However, at lower annotation levels, few variations were identified in aliquots treated with microbiome DNA enrichment methods, with NEBNext resulting in a more similar taxonomic profile to the untreated aliquots (Figure 7). These variations might be explained by the different enzymes and buffers used to lyse the host cells and to degrade the released DNA and by the different DNA purification procedures used; besides, variation in cell lysability and genome GC content of the different bacteria present in complex samples might result in the underestimation or overestimation of particular microbial taxa (Heravi et al., 2020). In this regard, the reduction of the relative abundance of some Gram-negative bacteria in milk and filter aliquots treated with microbial DNA enrichment methods is in line with the expected outcomes, though these results reflect possible biases in the microbial profile and in the AMR genes content.

The investigation of the microbiome performed in our study revealed a high proportion of sequences assigned to Gram-negative bacteria in both filter and milk aliquots, with taxonomic assignments depending on the sample type; our results are consistent with what emerged from some previous studies describing the microbiome of dairy products and dairy processing environments, as well as the minor proportion of sequences assigned to lactic acid bacteria compared to data referring to raw milk cheeses (Alexa Oniciuc et al., 2020). The role of commensal and pathogenic Gram-negative bacteria as vectors of AMR determinants in raw bulk tank milk has already been investigated and proven (Straley et al., 2006). In this context, regarding the AMR genes screening, this study shows that the milk production environment might be a rich reservoir of AMR determinants, with the detection of different genes conveying multi-drug resistance and resistance to rifampin, aminoglycosides, tetracyclines, sulfonamides, macrolides, lincosamides, streptogramins, and β-lactams. The possibility to uncover a wide range of determinants involved in AMR without the isolation and culture of bacteria must be considered particularly valuable, and especially relevant for food safety. However, as proved in this pilot study, choosing the right sample type and sample pre-treatment has a critical importance to correctly describe the resistome of food or food-related sample rich in host-DNA.

In summary, considering the different matrices, sequencing depths and microbial enrichment methods evaluated in this study, milk filters treated with MolYsis kit and sequenced to a depth of 60 M PE reads appeared to be the more suitable samples to obtain a deep taxonomic and AMR gene content profiling of the milk production environment evaluated; nevertheless, the increasing of costs and time spent manipulating the samples must be taken into account. The analysis of milk filters to investigate the microbiome and to identify the presence of foodborne pathogens or AMR genes has already proved to be a useful tool (Murphy et al., 2005; Sonnier et al., 2018; Dell’Orco et al., 2019). In-line filters can filter milk from approximately 150 cows, catching debris, large particles of organic material, and foreign objects. Therefore, the milk filters’ microbiome might include the presence of unexpected bacteria due to fecal contamination during the milking process and dispersal of cells from biofilms present in milking systems, thus reflecting the complex and rich milk production environment microbiome (Kim et al., 2018). Though retaining foreign objects, filter pores are too big to prevent bacteria to enter the bulk tank milk; as a consequence, the microbiome and the resistome of milk filters can be considered representative of the microbiome and the resistome of the milk production environment (Murphy et al., 2005).

To our knowledge, this is the first study to compare the effectiveness of sequencing depths and host-DNA removal kits on the characterization of the microbiome and the resistome of bulk tank milk and milk filters using a WMS approach. Due to the limit posed by the small number of samples, our study must be considered a pilot study; the results of our work are consistent with the results of previous researches applied on clinical samples and can assist in the design of WMS experiments on food matrices with a high content of host DNA, highlighting the importance of sample type, microbial enrichment method, and sequencing depth to profile the taxonomic and the AMR gene content.

## Data Availability Statement

The datasets presented in this study can be found in online repositories. The names of the repository/repositories and accession number(s) can be found at: https://www.ncbi.nlm.nih.gov/, PRJNA638876.

## Author Contributions

TC, FC, and SR contributed to conception and design of the study. SR and AD acquired the data. SR, AD, PC, and FC performed the data analysis and interpretation. SR and FC drafted the manuscript. All authors contributed to manuscript revision, read, and approved the submitted version.

### Conflict of Interest

The authors declare that the research was conducted in the absence of any commercial or financial relationships that could be construed as a potential conflict of interest.

## Footnotes

^1^https://github.com/lh3/seqtk

^2^https://www.bioinformatics.babraham.ac.uk/projects/fastqc
